# HIV Controllers Exhibit Effective CD8^+^ T Cell Recognition of HIV-1-Infected Non-activated CD4^+^ T Cells

**DOI:** 10.1016/j.celrep.2019.03.016

**Published:** 2019-04-02

**Authors:** Blandine Monel, Annmarie McKeon, Pedro Lamothe-Molina, Priya Jani, Julie Boucau, Yovana Pacheco, R. Brad Jones, Sylvie Le Gall, Bruce D. Walker

**Affiliations:** 1 Ragon Institute of Massachusetts General Hospital, Massachusetts Institute of Technology, and Harvard University, Cambridge, MA 02139, USA; 2 Howard Hughes Medical Institute, Chevy Chase, MD 20815, USA; 3 Center for Autoimmune Diseases Research (CREA), School of Medicine and Health Sciences, Universidad del Rosario, Bogotá, Colombia; 4 Division of Infectious Diseases, Weill Cornell Medicine, New York, NY 10065, USA; 5 Institute for Medical Engineering and Science, Massachusetts Institute of Technology, Cambridge, MA 02139, USA

**Keywords:** HIV cure, cytotoxic T lymphocytes, HLA, elite controllers, HIV, perforin, granzyme, immunologic synapse

## Abstract

Even with sustained antiretroviral therapy, resting CD4^+^ T cells remain a persistent reservoir of HIV infection, representing a critical barrier to curing HIV. Here, we demonstrate that CD8^+^ T cells recognize infected, non-activated CD4^+^ T cells in the absence of *de novo* protein production, as measured by immune synapse formation, degranulation, cytokine production, and killing of infected cells. Immune recognition is induced by HLA-I presentation of peptides derived from incoming viral particles, and recognition occurred either following cell-free virus infection or following cell-to-cell spread. CD8^+^ T cells from HIV controllers mediate more effective immune recognition than CD8^+^ T cells from progressors. These results indicate that non-activated HIV-infected CD4^+^ T cells can be targeted by CD8^+^ T cells directly after HIV entry, before reverse transcription, and thus before the establishment of latency, and suggest a mechanism whereby the immune response may reduce the size of the HIV reservoir.

## Highlights

• Non-activated CD4^+^ T cells are permissive for HIV entry but not productively infected
• CD8^+^ T cells from HIV controllers recognize HIV^+^ non-activated CD4^+^ T cells
• Antigens from incoming viral particles are presented to CD8^+^ T cells through HLA-I
• CD8^+^ T cell recognition leads to the death of the HIV^+^ non-activated CD4^+^ T cells

## Graphical Abstract

**Figure:**
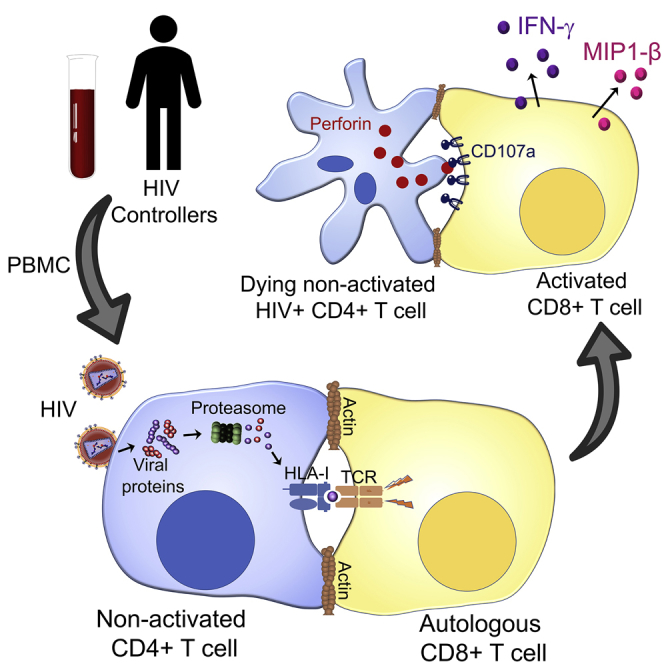

## Teaser

The cure for HIV is not achievable due to HIV reservoirs, mostly in resting CD4^+^ T cells. Monel et al. show that CD8^+^ T cells from HIV controllers are able to establish immunological synapses with HIV^+^ resting CD4^+^ T cells, leading to IFN-γ, MIP1-β production, degranulation, and the elimination of the target cells.

## Introduction

Despite the ability of combination antiretroviral therapy (cART) to reduce plasma viremia to undetectable levels, treatment does not lead to viral eradication in HIV-infected persons, committing them to lifelong therapy. The inability to eradicate HIV is predominantly due to a reservoir of resting CD4^+^ T cells non-productively infected by HIV (Bukrinsky et al., 1991, Chun et al., 1997, Finzi et al., 1997, Massanella and Richman, 2016, Siliciano et al., 2003, Soriano-Sarabia et al., 2014). Resting CD4^+^ T cells are permissive for HIV entry (Tilton et al., 2014), but once inside, cytoplasmic host restriction factors such as SAMHD1 can impede the reverse transcription of viral RNA into cDNA (Baldauf et al., 2012, Descours et al., 2012), leading to abortive infection. The resulting cDNA fragments are sensed by interferon-γ (IFN-γ)-inducible protein (IFI-16) and induce pyroptosis, leading to the production of pro-inflammatory cytokines and subsequent CD4^+^ T cell depletion (Doitsh et al., 2010, Doitsh et al., 2014, Monroe et al., 2014).

Although HIV infection of resting CD4^+^ T cells is mostly abortive (Doitsh et al., 2010, Tilton et al., 2014), reverse transcription can occasionally be completed and the viral cDNA imported into the nucleus, resulting in either pre- or post-integration latency (Chavez et al., 2015, Pan et al., 2013, Zhou et al., 2005) without an intermediate phase of productive infection (Chavez et al., 2015, Vatakis et al., 2009). Pre-integration latency occurs when viral cDNA is blocked in the nucleus of a resting cell without being able to integrate into the host chromosome (Petitjean et al., 2007, Pierson et al., 2002, Sloan and Wainberg, 2011); subsequent integration can occur after cellular activation, leading to productive infection (Thierry et al., 2015). Post-integration latency is the most stable form of latency in which viral cDNA is efficiently integrated but leads to no or few viral transcripts (Dahabieh et al., 2015, Hakre et al., 2012, Stevenson, 1997). These cells contain a viral genome that can be reactivated by different factors, leading to the production of new infectious particles (Bruner et al., 2015, Chun et al., 1997, Chun et al., 1998, Davey et al., 1999, Ho et al., 2013, Laird et al., 2015, Wong et al., 1997). Both forms of latency contribute to a reservoir of resting infected cells that are presumed to be invisible to HIV-specific CD8^+^ T cell responses. The persistence of this HIV reservoir is a major obstacle to current cure efforts (Archin and Margolis, 2014, Katlama et al., 2013, Massanella et al., 2013, Pitman et al., 2018, Siliciano and Siliciano, 2013).

Killing HIV-infected resting CD4^+^ T cells early after viral entry before the reverse transcription step would abrogate both abortive and latent infection and would thus help to decrease CD4^+^ T cell depletion and inflammation, and could affect the size of the latent viral reservoir. It has been shown that HIV Gag and Pol-specific CD8^+^ T cell lines recognize peptides from incoming particles after HIV entry into activated CD4^+^ T cells (Payne et al., 2010, Kløverpris et al., 2013), and at least one study indicates that resting cells may also be targeted (Buckheit et al., 2013), although the role of antigen processing, restricting human leukocyte antigen (HLA) alleles, and synapse formation in the observed elimination of infected cells was not evaluated. We tested the hypothesis that HIV-specific CD8^+^ T cells from HIV controllers, a very small proportion of the HIV-infected population who manage to spontaneously control viral replication and maintain stable CD4^+^ T cell counts without the need for antiretroviral therapy (Carrington and Walker, 2012, Deeks and Walker, 2007, Walker and Yu, 2013), could recognize and kill non-activated, infected CD4^+^ T cells due to the recognition of processed viral proteins following viral entry, without requiring productive infection. We used a combination reporter virus system that allowed us to sensitively and specifically track the kinetics of infection of resting CD4^+^ T cells after viral entry and before any *de novo* viral protein production. We show that CD8^+^ T cells from HIV controllers readily establish functional synapses with non-activated infected CD4^+^ T cells, leading to HLA class I-restricted degranulation, cytokine production, and target cell death, and does not require reverse transcription, indicating that *de novo* viral protein production is not needed. Moreover, we show that cell-cell transmission also sensitized cells to HIV-specific CD8^+^ T cell recognition, before viral reverse transcription occurs. This response is significantly more potent in HIV controllers than in progressors, suggesting a mechanism whereby the immune response may influence the size of the HIV reservoir.

## Results

### HIV Infection of Primary Non-activated CD4^+^ T Cells

Direct HIV infection of non-activated CD4^+^ T cells leads predominantly to abortive infection and to a lesser extent, latent infection, which renders cells largely invisible to HIV-specific CD8^+^ T cells (Pan et al., 2013, Tilton et al., 2014). Since incoming virions can sensitize activated CD4^+^ T cells for recognition by CD8^+^ T cells (Buseyne et al., 2001, Kløverpris et al., 2013, Payne et al., 2010), we first sought to confirm whether resting CD4^+^ T cells would likewise be permissive for HIV entry, as previously shown (Tilton et al., 2014), and to determine whether these cells could be recognized by CD8^+^ T cells pre-integration and thus before possible abortive infection or establishment of latent infection.

To assess the ability of non-activated CD4^+^ T cells to become infected with HIV, we used a combination reporter virus system that allowed for discrimination between viral entry into the cytoplasm and subsequent *de novo* virion production in the infected cell (Tilton et al., 2014). Resting CD4^+^ T cells were infected with HIV containing β-lactamase fused to HIV Vpr (Vpr-βlam). Viral entry was detected by pre-labeling cells with a fluorescence resonance energy transfer (FRET) cytoplasmic substrate (coumarin cephalosporin fluorescein, a fluorescent beta-lactamase substrate [CCF2-AM]) composed of a hydroxycoumarin donor conjugated to a fluorescein acceptor via a β-lactam ring. Cleavage of the β-lactam ring is mediated via the β-lactamase protein carried by the incoming virus, inducing an emission shift that allows for the colorimetric detection of viral entry into the cell by flow cytometry. *De novo* HIV protein production was detected by means of HIV long terminal repeat (LTR)-driven GFP expression (Cavrois et al., 2002, Tilton et al., 2014).

Using this system, we assessed viral entry and levels of productive infection, comparing activated to non-activated CD4^+^ T cells from healthy donors. The activation status of live CD3^+^CD4^+^ T cells in whole peripheral blood mononuclear cells (PBMCs) was assessed *ex vivo* by flow cytometry by analyzing the expression of CD25 and CD69, inducible cell surface glycoproteins acquired during lymphocyte activation. In the absence of exogenous stimulation, CD4^+^ T cells within the PBMCs were quiescent, but were readily activated by incubation with CD3/CD28 beads for 2 days. A representative experiment is shown in Figure S1A. Of note, the activation status was similar when CD4^+^ T cells were first isolated from PBMCs (data not shown).

**Figure 1:**
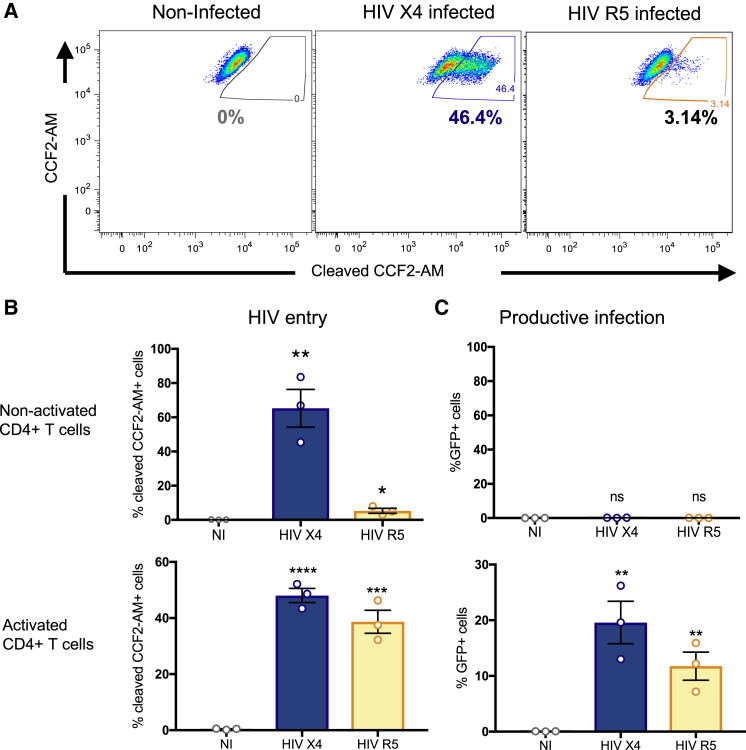
HIV Infection in Primary Non-activated CD4^+^ T Cells (A) Non-activated CD4^+^ T cells were infected for 2 h with NL4.3 X4 (HIV X4, blue) or R5 virus (HIV R5, yellow) carrying the fusion protein Vpr-β-lactamase and an internal ribosome entry site (IRES)-GFP cassette. HIV entry was determined 2 h later by incubation with an FRET-β-lactamase substrate, where cleaved substrate^+^ cells represent HIV^+^ cells. A representative experiment is shown. (B) Composite infection data as in (A) for non-activated (top) and activated (bottom) CD4^+^ T cells, expressed as means ± SDs for three independent experiments from three different individuals, each performed in duplicate. (C) The percentage of cells expressing GFP, representing productive infection, was measured 48 h post-infection in non-activated CD4^+^ T cells (top) and activated CD4^+^ T cells (bottom). The results are expressed as means ± SDs for three independent experiments from three different individuals, each performed in duplicate. Statistical significance was calculated using an unpaired t test relative to the non-infected (NI) condition. ^∗^p < 0.05, ^∗∗^p < 0.01, ^∗∗∗^p < 0.001, and ^∗∗∗∗^p < 0.0001. See also Figures S1 and S2.

Two hours following infection, activated and non-activated CD4^+^ T cells were assessed for viral entry, as evidenced by β-lactamase-mediated cleavage and fluorescence of the cytoplasmic substrate. Non-activated (CD25^−^, CD69^−^) CD4^+^ T cells were highly permissive to entry by X4-tropic HIV (Figure 1A), with viral entry detected in 65% ± 11% of resting CD4^+^ T cells at the multiplicity of infection used (Figure 1B, top). The entry of R5 tropic viruses was also detected, but to a lesser extent (5% ± 1% of resting CD4^+^ T cells), consistent with lower C-C chemokine receptor type 5 (CCR5) expression on the resting CD4^+^ T cells (Figures 1B, bottom, and S1B). Similar levels of infection were observed when non-activated CD4^+^ T cells were first isolated from PBMCs (data not shown). To be certain that the cleaved substrate corresponded to viral entry, a virus missing the envelope (HIV ΔEnv) and a fusion-defective virus (HIV X4 Env-F522Y) were used as controls (Figure S2). Quantification of GFP expression in CD4^+^ T cells 2 days later revealed that most of the non-activated HIV-exposed CD4^+^ T cells remained non-productively infected, contrary to activated CD4^+^ T cells (Figure 1C). These results are consistent with previous reports (Haqqani et al., 2015, Tilton et al., 2014) and further suggest that most of the directly infected non-activated CD4^+^ T cells remain non-productively infected during the period observed.

### Recognition of HIV^+^ Non-activated CD4^+^ T Cells by HIV-Specific CD8^+^ T Cells

**Figure 2:**
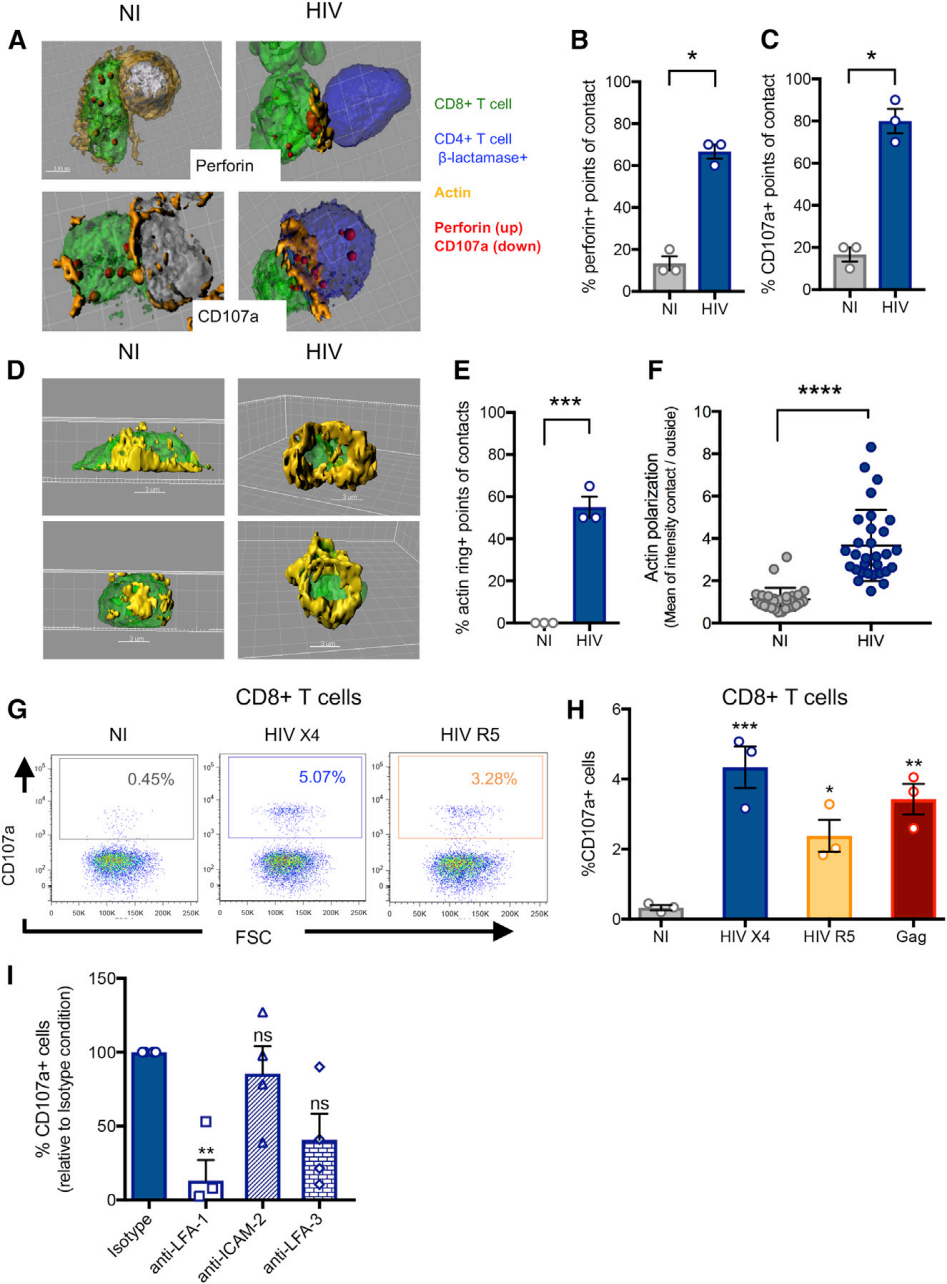
Recognition of HIV^+^ Non-activated CD4^+^ T Cells by Autologous HIV-Specific CD8^+^ T Cells Two hours after a Vpr-βlam infection assay, the primary non-activated HIV^+^ CD4^+^ T cells (blue) from an elite controller (EC) were co-cultured for 30 min with an autologous Gag-specific CD8^+^ T cell line (green), fixed, and stained for actin with phalloidin (orange) and perforin (red) or CD107a (red), and cell contacts analyzed by confocal microscopy. A compilation of z stacks was acquired for each cell contact and analyzed with IMARIS software. NI, non-infected; HIV, HIV infected. (A) 3D reconstructions of two representative experiments with non-infected cells (left) or infected cells (right) are shown with perforin staining (top) or CD107a staining (bottom). (B and C) The percentage of points of contact displaying fluorescent signal for perforin (B) or CD107a (C) is quantitated and shown. Means ± SDs for three independent experiments using cells coming from three different HIV controllers are shown; statistics were calculated by unpaired t test. ^∗^p < 0.05. (D) For each cell contact, the actin signal was analyzed and quantified with IMARIS software in 3D. Two representative experiments are shown, demonstrating actin rings only in the HIV-infected condition. (E) The percentage of points of contact displaying actin rings is calculated for both conditions and shown as means ± SDs for three independent experiments with cells coming from three different HIV controllers. Statistics were calculated by unpaired t test. (F) Actin polarization on CD8^+^ T cells toward the point of contact was quantified by measuring the ratio of actin fluorescent signal at the point of contact versus the rest of the cell. The means ± SDs for three independent experiments with 10 cell contacts analyzed per experiment with cells coming from three different HIV controllers; statistics were calculated by unpaired t test. (G) CD107a staining gated on live CD3^+^CD8^+^ cells revealed the degranulation of CD8^+^ T cells in response to autologous HIV X4 infected cells (blue) or HIV R5 infected cells (yellow), compared to Gag peptide-sensitized target cells (red). A representative experiment is shown. (H) The means ± SDs for three independent experiments with cells coming from one HIV controller are represented. Statistics were calculated by an ANOVA multiple comparison test relative to the non-infected condition. ^∗^p < 0.05, ^∗∗^p < 0.01, and ^∗∗∗^p < 0.001. (I) Antibodies to LFA-I, ICAM-2, and LFA-3 were used in an experiment similar to (G) with HIV X4. The means ± SDs for four independent experiments with cells coming from four different HIV controllers are shown, and statistics were calculated with a one-way ANOVA multiple comparison test relative to the isotype control. ^∗∗^p < 0.01; ns, not statistically significant. See also Video S1 and Figures S3 and S4.

We next tested whether an HIV Gag-specific CD8^+^ T cell line derived by stimulating PBMCs from an HIV controller with a Gag peptide pool could recognize these non-productively infected CD4^+^ T cells. We first examined the formation of functional immunological synapses by fluorescence microscopy. A cytolytic immunological synapse requires recognition of the viral peptide:major histocompatibility complex (MHC) by the T cell receptor (TCR) and polarization of adhesion molecules including LFA-1 and actin toward the point of contact to form a ring with central clearance, through which cytolytic granules are released (Ritter et al., 2015, Stinchcombe and Griffiths, 2003). To determine whether immune synapse formation was occurring, HIV-infected non-activated CD4^+^ T cells (defined by the expression of the β-lactamase cleaved substrate) were co-cultured for 30 min with an autologous HIV Gag-specific CD8^+^ T cell line 3 h post-HIV entry, a time point shown above to lack viral protein production. Ten contacts between CD4^+^ T cells and CD8^+^ T cells from three independent experiments were analyzed by confocal microscopy and are represented with 3D reconstructions (Figure 2A), showing the presence of perforin (upper images) or the degranulation marker CD107a (lower images) at the point of contact. Quantitative image analysis revealed higher perforin (Figure 2B) and CD107a (Figure 2C) expression at points of contact with HIV^+^ CD4^+^ T cells (average 66% ± 3% and 80% ± 6% of points of contact were positive for perforin or granzyme, respectively) compared to non-infected CD4^+^ T cells (13% ± 3% and 17% ± 3%, respectively). The observed background likely represents random localization of the cytolytic granules near the point of contact. The presence of immune synapses was supported by the observation of an actin ring (blinded counting) for 55% ± 5% of the contacts between HIV^+^ CD4^+^ T cells and the autologous CD8^+^ T cells compared to 0% for non-infected CD4^+^ T cells (Figures 2D and 2E), and higher actin polarization toward the point of contact (Figure 2F) automatically determined by the ratio of actin fluorescence signal at the point of contact versus the rest of the cell with Imaris software. Video S1 shows the rotation of a 3D synapse indicating the polarization and the actin ring at the synapse. These results show that an HIV-specific CD8^+^ T cell line can establish immune synapses with autologous HIV^+^ non-activated CD4^+^ T cells following viral entry, in the absence of *de novo* viral protein production.

Video S1. Rotation of a 3D Reconstruction of an Immunological Synapse, Related to Figure 2 Synapse between an HIV+ non-activated CD4+ T cell (blue) and an HIV-specific CD8+ T cell (orange) with an actin ring (green).

We next evaluated effector functions elicited by the CD8^+^ T cells upon the recognition of non-productively HIV-infected CD4^+^ T cells using flow cytometry. Non-activated PBMCs from an HIV controller were infected with HIV (X4 or R5 Vpr-βlam^+^) for 2 h, washed to remove free virus, and then incubated at 37°C for 5 h in the presence of anti-CD107a antibody. HIV entry was documented with a parallel aliquot of cells using the Vpr-βlam assay and flow cytometry gating on the CD4^+^ T cells. At the end of the 5-h incubation, the CD8^+^ T cell response in whole PBMCs was determined by flow cytometry by measuring CD107a expression on live CD3^+^CD8^+^ T cells. Non-activated CD4^+^ T cells infected with either X4 or R5 virus using 500 ng of viral p24 based on dose-response experiments (Figure S3) elicited significant CD8^+^ T cell degranulation compared to the non-infected condition (p < 0.001 and p < 0.05, respectively) (Figures 2G and 2H), indicating that the immunological synapses led to the functional engagement of CD8^+^ T cells. Since R5 viral entry rates were relatively low (approximately 5% of non-activated CD4^+^ cells compared to 60% with X4 virus; Figure 1B), these results suggest that only a few HIV^+^ CD4^+^ T cells are required to induce a CD8^+^ T cell response in this assay, irrespective of co-receptor usage, since we observed only a 2-fold higher CD8^+^ T cell response with X4 than R5 HIV infection despite a 12-fold higher HIV entry.

We further evaluated intracellular expressions of IFN-γ, tumor necrosis factor α (TNF-α), and perforin in CD8^+^ T cells and observed similar polyfunctionality profiles in CD8^+^ T cells responding to either Gag peptide pool stimulation or to infected, non-activated CD4^+^ T cells (Figure S4). We conclude that non-productively HIV-infected CD4^+^ T cells, before viral protein production, elicit similar CD8^+^ T cell responses to autologous cells loaded with pooled Gag peptides.

The above results suggest that autologous CD8^+^ T cells can form immunological synapses and degranulate and release cytokines in response to non-productively HIV-infected resting CD4^+^ T cells. To assess the relation between synapse formation and degranulation, we inhibited the formation of immunological synapses using antibodies against adhesion molecules involved in this interaction (LFA-1, intercellular adhesion molecule 2 [ICAM-2], LFA-3). Consistent with our hypothesis, blocking LFA-I, the main adhesion molecule involved in immunological synapse formation (Dustin and Shaw, 1999, Grakoui et al., 1999), resulted in decreased degranulation of CD8^+^ T cells (Figure 2I). Blocking LFA-3 or ICAM-2 did not reduce the CD8^+^ T cell response, suggesting that these molecules may be less important to the recognition of infected, non-activated CD4^+^ T cells.

### CD8^+^ T Cell Recognition of Non-activated C4^+^ T Cells following Cell-Cell Transmission

**Figure 3:**
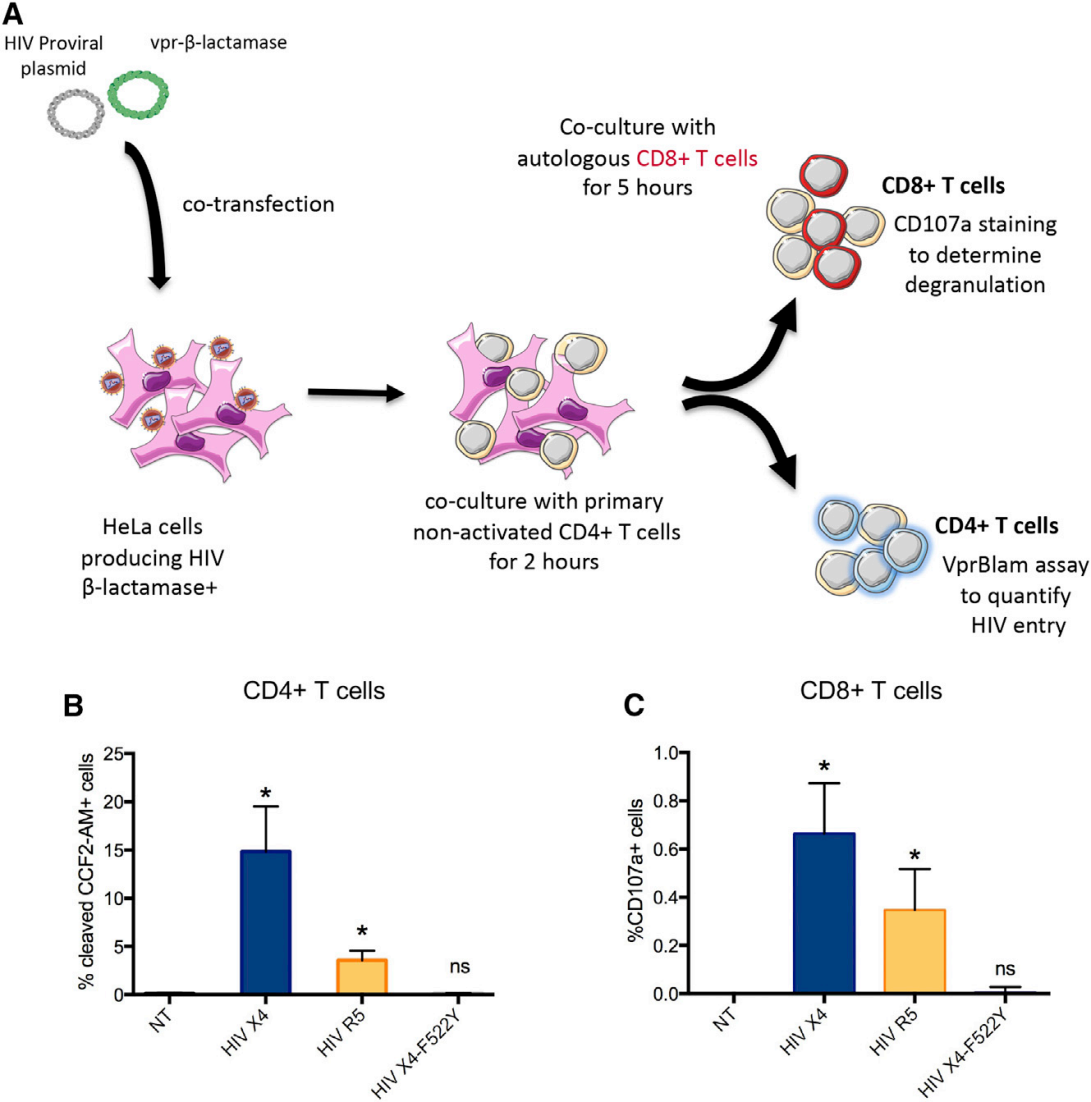
HIV Cell-to-Cell Transmission in Non-activated CD4^+^ T Cells and Autologous CD8^+^ T Cell Recognition HeLa cells transfected with HIV Vpr-βlam plasmids were co-cultured for 2 h at 37°C with primary non-activated CD4^+^ T cells from an HIV controller, and then the CD4^+^ T cells were separated and put in co-culture with autologous primary bulk CD8^+^ T cells for 5 h. (A) Schematic representation of the experiment. (B) A Vpr-βlam assay was conducted on the non-activated CD4^+^ T cells after co-culture with transfected HeLa cells to assess HIV entry by HIV X4 (blue), HIV R5 (yellow), or the control fusion defective virus HIV X4-F522Y (gray). (C) CD107a staining of CD8^+^ T cells as analyzed by flow cytometry. The means ± SDs for three independent experiments with cells from three different HIV controllers are represented, and statistics were calculated with an ANOVA multiple comparison test relative to the non-transfected (NT) condition. ^∗^p < 0.05.

The above results were obtained using cell-free infection with a high viral inoculum. To further confirm our results under more physiologic conditions, we established a cell-to-cell transmission assay to simulate what would be expected to occur in tissues *in vivo* (Monel et al., 2012, Sourisseau et al., 2007). HeLa cells were transfected with viral plasmids encoding Vpr-βlam viruses: HIV X4, HIV R5, or an X4 virus containing a mutation in the envelope (F522Y) preventing viral fusion with the cellular membrane, but not the interaction with the receptor and co-receptor (Bergeron et al., 1992, Clavel et al., 1989). HeLa cells were subsequently co-cultured with non-activated, CCF2-AM labeled uninfected CD4^+^ T cells for 2 h. A Vpr-βlam assay was performed on an aliquot of the CD4^+^ T cells, confirming cell-to-cell transmission and viral entry. The remaining CD4^+^ T cells were co-cultured with primary autologous bulk CD8^+^ T cells for 5 h in the presence of CD107a antibody to analyze CD8^+^ T cell degranulation (Figure 3A). In this system, primary, non-activated CD4^+^ T cells were permissive to HIV X4 and HIV R5 entry by cell-to-cell transmission from transfected HeLa cells, but were not infected by the fusion-defective virus F522Y (Figure 3B). Furthermore, viral entry into resting CD4^+^ T cells in this assay induced CD8^+^ T cell degranulation directly *ex vivo* using unstimulated PBMCs from HIV-infected donors (Figure 3C). The relatively low levels of specific degranulation are consistent with expectations, given the frequency of HIV-specific effector CD8^+^ T cells in PBMCs (data not shown). These data confirm the previous observations of CD8^+^ T cell recognition of non-productively HIV-infected CD4^+^ T cells, in this case following cell-cell transmission.

### Incoming Viral Particles Present Viral Peptides to CD8^+^ T Cells

**Figure 4:**
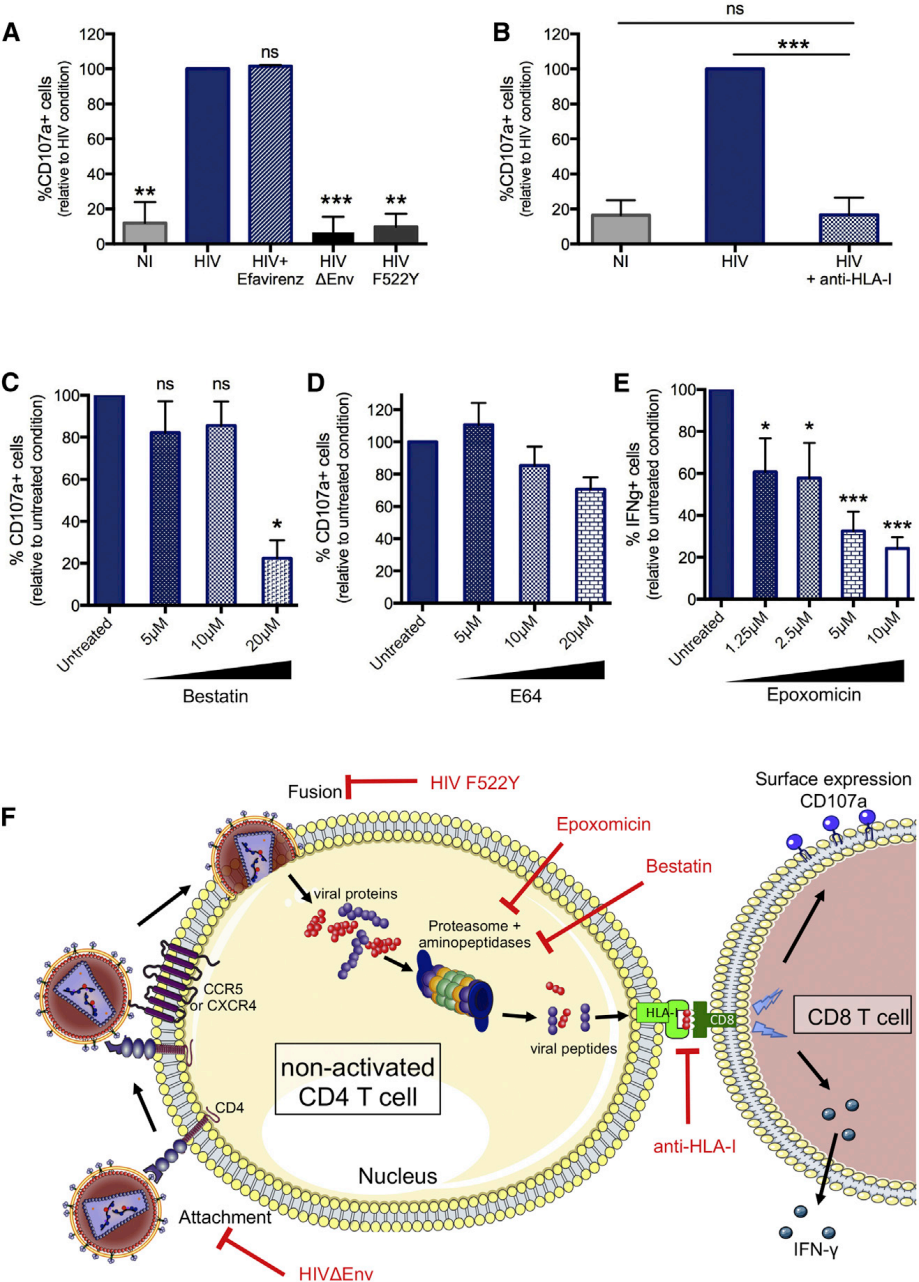
CD8^+^ T Cell Response Induced by the Presentation of Viral Peptides from Incoming HIV Particles in Non-activated CD4^+^ T Cells through the HLA-I Molecule (A) The CD8^+^ T cell response to HIV^+^ non-activated CD4^+^ T cells was evaluated by flow cytometry with CD107a staining without HIV Env-CD4 and co-receptor interactions (HIV ΔEnv), without HIV entry into CD4^+^ T cells (HIV F522Y), or in the presence of a reverse transcription inhibitor (efavirenz) and compared to HIV infection alone (HIV). (B) The importance of the HLA-I molecule in the CD8^+^ T cell response was evaluated by blocking the HLA-I molecule with the anti-class I antibody (clone W6/32), and measuring degranulation. (C–E) The role of the antigen-processing pathway in CD8^+^ T cell recognition was evaluated by measuring degranulation by flow cytometry. This was done by blocking the aminopeptidases with bestatin (C), the cysteine proteases with E64 (D), and the proteasome with epoxomicin (E). (F) Schematic representation of the required steps from viral entry to antigen presentation in a non-activated CD4^+^ T cell to induce a CD8^+^ T cell response. Means ± SDs for three independent experiments with cells coming from three different HIV controllers are represented. Statistics were calculated with ANOVA multiple comparison tests relative to the HIV condition for (A), each column relative to every other column for (B), and relative to the untreated condition for (C)–(E). ^∗^p < 0.05, ^∗∗^p < 0.01, and ^∗∗∗^p < 0.001. Regarding epoxomicin, the drug induced CD107a surface expression on CD8^+^ T cells by itself through an unknown mechanism, but in the absence of HIV peptide stimulation or HIV exposure (data not shown). For this reason, IFN-γ staining was used.

Previous studies have shown that activated simian immunodeficiency virus-negative (SIV^−^) and HIV-infected CD4^+^ T cells can be recognized by CD8^+^ T cells in response to the presentation of viral peptides derived from incoming particles and presented by HLA class I molecules (Kløverpris et al., 2013, Payne et al., 2010, Buseyne et al., 2001). We next investigated whether this was also the case for non-activated HIV^+^ CD4^+^ T cells in which activities of antigen-processing enzymes are lower than in activated CD4^+^ T cells (J.B., unpublished data). To address this question, cells were infected with a wild-type (WT) HIV X4 virus, a fusion-defective mutant (HIV F522Y), or a virus rendered non-infectious by the lack of the envelope protein (HIV ΔEnv), and the experiment was conducted in the presence or absence of the reverse transcriptase inhibitor efavirenz. As shown in Figure 4A, degranulation in response to fusion-defective virus was reduced to background levels. Thus, virus entry into the cytoplasm, and not potential stress signaling caused by Env-receptor and co-receptor interactions, was absolutely necessary to induce an HIV-specific CD8^+^ T cell response. We also determined that viral reverse transcription was not required to trigger the CD8^+^ T cell degranulation, as shown by the same level of CD107^+^ CD8^+^ T cells with or without efavirenz (Figure 4A).

Based on these data, we hypothesized that CD8^+^ T cell responses are triggered shortly after viral entry into the cytoplasm of the target cell, suggesting a role for incoming, as opposed to *de novo* transcribed, viral particles. Anti-HLA class I antibody blockade during the following 5-h incubation period post-infection resulted in a marked decrease in CD8^+^ T cell responses (Figure 4B), indicating that the CD8^+^ T cell degranulation observed in response to HIV^+^ non-activated CD4^+^ T cells is driven by HLA class I presentation of viral peptides entering the cytoplasm after viral fusion.

To more precisely define the involvement of antigen processing in the recognition of non-activated cells, we blocked enzymatic aminopeptidase activities with bestatin, cysteine cathepsin activities with E64, and the proteasome with epoxomicin (Bogyo and Wang, 2002, Groll and Huber, 2004, Mathé, 1991, Vaithilingam et al., 2013). Inhibition of aminopeptidases with bestatin or blocking of proteasome activities, but not blocking of cysteine cathepsin activities, resulted in a decrease in CD8^+^ T cell responses to HIV^+^ non-activated CD4^+^ T cells (Figures 4C–4E). The results from these pharmacological inhibition experiments support that antigen processing is a prerequisite for CD8^+^ T cell recognition of non-productively infected CD4^+^ T cells. These data suggest that HIV fusion into the cytoplasm, HIV degradation by aminopeptidases and proteasomes, but not reverse transcription, are required for antigen processing of incoming viral particles and presentation by HLA class I (Figure 4F).

### Killing of HIV^+^ Non-activated CD4^+^ T Cells by HIV-Specific CD8^+^ T Cells

**Figure 5:**
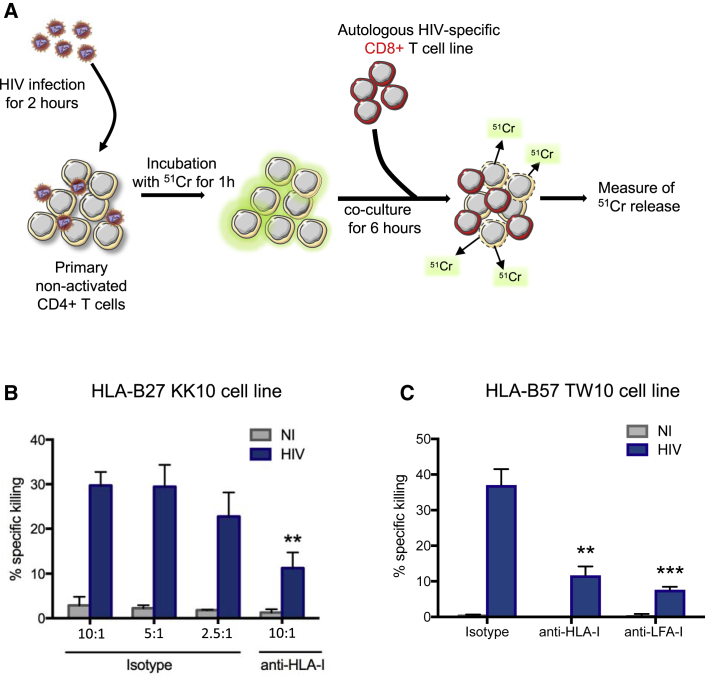
Killing of HIV^+^ Non-activated CD4^+^ T Cells by Autologous HIV-Specific CD8^+^ T Cells (A) Schematic illustration of the experiment. (B and C) Non-activated CD4^+^ T cells from HIV controllers were infected with HIV-Vpr-βlam^+^ for 2 h and then incubated with ^51^Cr for 1 h. The cells were then co-cultured with autologous KK10-specific (B) or TW10-specific CD8^+^ T cell lines (C) at the indicated effector:target cell ratios for 6 h. Blocking antibodies against HLA-I or LFA-I were added before co-culture, as indicated. The release of ^51^Cr in the supernatant was measured, and the percentage of specific killing was calculated as [(mean experimental cpm − mean spontaneous cpm)/(mean maximum cpm − mean spontaneous cpm)] × 100. Means ± SDs for two independent experiments in duplicate with cells coming from two different HIV controllers are represented, and statistics were calculated with a two-way ANOVA multiple comparison test relative to 1:10 isotype condition. ^∗∗^p < 0.01 and ^∗∗∗^p < 0.001.

The above studies show that HIV-infected, non-activated CD4^+^ T cells trigger CD107a expression, but do not directly demonstrate that these cells are killed. To address this, we performed a chromium release assay using HIV^+^ non-activated CD4^+^ T cells (Chen et al., 2012) shortly after viral entry (Figure 5A). Release of ^51^Cr into the supernatant was quantified, revealing direct killing by autologous HIV-specific CD8^+^ T cell lines at multiple effector-to-target ratios (Figures 5B and 5C). Furthermore, killing was dramatically decreased in the presence of an anti-HLA-I antibody compared to isotype antibody controls (Figure 5B) and in the presence of anti-LFA1 antibody, confirming the requirement for functional immunological synapses for efficient killing (Figure 5C). We conclude that HIV-specific CD8^+^ T cells are able to not only degranulate upon recognition of HIV^+^ non-activated CD4^+^ T cells within hours of viral fusion but also kill these cells.

### CD8^+^ T Cell Responses to Non-productively Infected CD4^+^ T Cells Are Preferentially Detected in HIV Controllers

**Figure 6:**
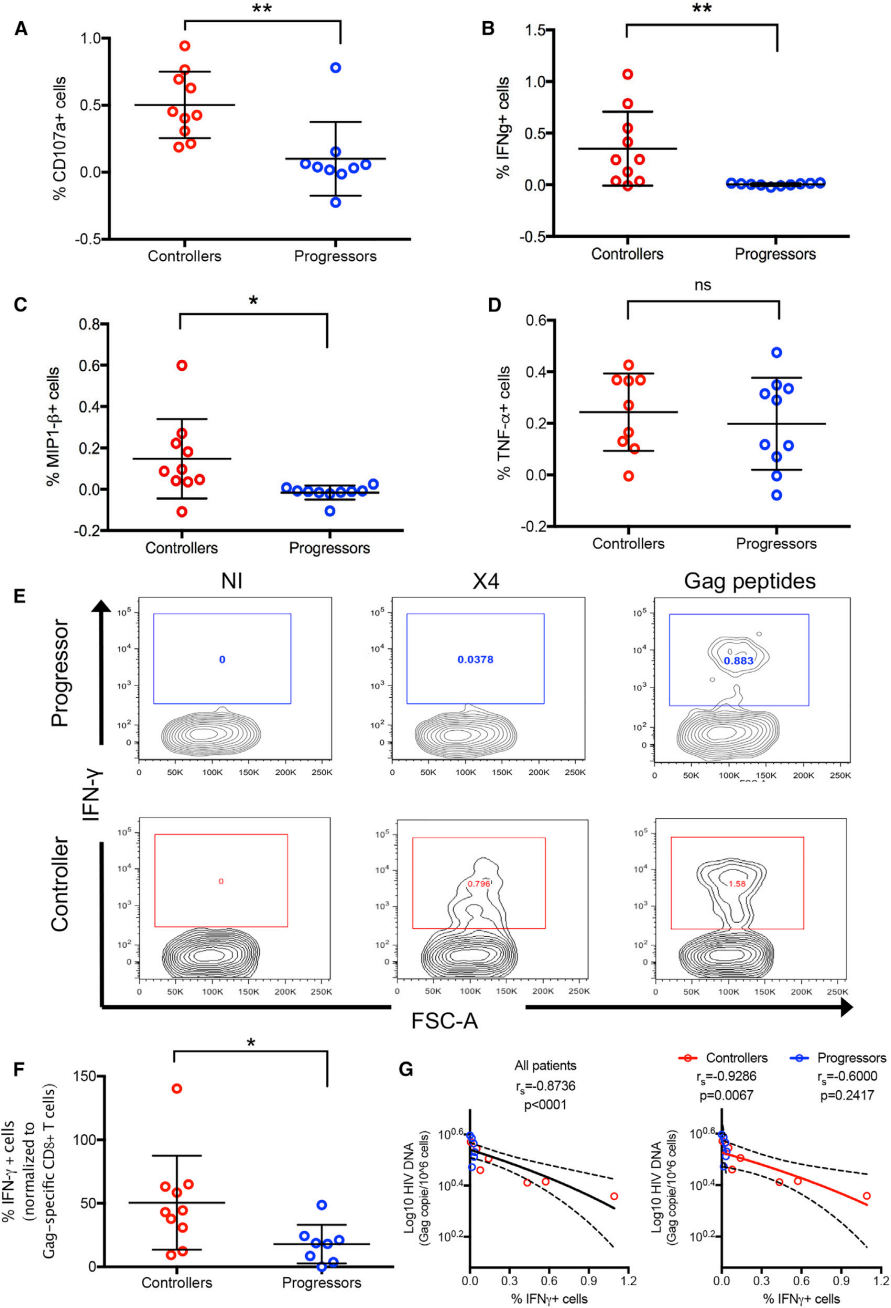
The CD8^+^ T Cell Response to HIV^+^ Non-activated CD4^+^ T Cells Participates in HIV Control (A–D) CD8^+^ T cell responses from HIV controller (red) or progressor (blue) patients (all HLA-B^∗^27 or B^∗^57, and n = 10 patients for both groups) in the context of whole non-activated PBMCs were measured by (A) CD107a expression, (B) intracellular IFN-γ expression, (C) intracellular MIP-1β expression, and (D) intracellular TNF-α expression after 2 h of HIV infection and 5 h of incubation. Results are shown following background subtraction. Statistical differences were assessed using PRISM software and an unpaired t test. ^∗^p < 0.05, ^∗∗^p < 0.01, and ns, not statistically significant. (E) CD8^+^ T cell response to Gag pool peptides (final concentration 1 μg/mL/peptide) was measured for each patient by intracellular IFN-γ staining. A representative experiment is shown for an HIV progressor (top) and for an HIV controller (bottom). (F) The CD8^+^ T cell response to HIV^+^ non-activated CD4^+^ T cells expressed relative to the Gag pool peptide response. (G) The HIV DNA reservoir was measured by droplet PCR, and the Spearman correlation between the log_10_ of Gag copies/10^6^ non-activated CD4^+^ T cells and the CD8^+^ T cell IFN-γ response is shown for all of the patients (left, n = 12 patients) and by sub-groups (right, n = 6 patients for each group). See also Table 1 and Figures S5 and S6.

We next assessed whether the recognition of HIV^+^ non-activated CD4^+^ T cells by HIV-specific CD8^+^ T cells was enhanced in HIV controllers compared to HIV progressors by comparing CD8^+^ T cell responses in 10 HIV controllers and 10 progressors, each expressing at least one protective allele (HLA-B^∗^27 or HLA-B^∗^57; Table 1). As described above, non-activated PBMCs from these individuals were infected with HIV X4, and the CD8^+^ T cells responses were analyzed by flow cytometry 5 h later using unfractionated PBMCs. Only CD8^+^ T cells from HIV controllers recognized HIV^+^ non-activated CD4^+^ T cells, as shown by degranulation (p = 0.004; Figure 6A), IFN-γ production (p = 0.007; Figure 6B), and macrophage inflammatory protein 1β (MIP-1β) production (p = 0.016; Figure 6C). Of note, there were no differences in the production of TNF-α (Figure 6D), perforin, or granzyme B (Figures S5A and S5B) by the Gag-specific CD8^+^ cells from HIV controllers and HIV progressors.

**Table 1:** Characteristics of HIV-1-Infected Study Participants

ID	HLA-A1	HLA-A2	HLA-B1	HLA-B2	HLA-CW1	HLA-CW2	ARV Therapy Status	CD4 (Number/mm^3)^	HIV VL (Copies/mL)
CP1	01:01	31:01	40:01	57:02	03:04	06:02	on therapy 10 y interrupted	953	≤50
CP2	02:01	02:01	27:02	51:01	02:02	02:02	on therapy 1 y	313	≤50
CP3	01:01	24:02	35:03	57:01	07:01	12:03	on therapy 1 y	575	95
CP4	02:01	24:02	27:05	27:05	01:02	02:02	on therapy 1 y	713	197
CP5	02:01	02:01	27:05	44:02	01:02	02:02	on therapy <1 y	587	17,583
CP6	01:01	68:02	14:02	57:01	06:02	08:02	on therapy 3 y	421	≤50
CP7	01:01	32:02	08:01	27:05	01:02	07:01	on therapy 3 y	1,208	≤50
CP8	01:02	11:01	27:06	81:01	03:04	08:04	on therapy 4 y	419	≤50
CP9	01:01	23:01	44:03	57:01	04:01	06:02	on therapy 1 y	408	75
CP10	01:01	03:01	35:01	57:01	06:02	08:02	on therapy 2 y	521	≤50
EC1	01:01	02:01	51:01	57:01	06:02	14:02	off therapy	797	≤50
VC2	02:01	30:01	13:02	57:01	06:02	06:02	ARV naive	579	1,470
VC3	32:01	32:01	27:05	44:02	01:02	05:01	off therapy	1,239	189
VC4	02:06	25:01	27:05	37:01	03:03	06:02	off therapy	352	51
EC5	01:01	11:01	27:05	35:03	02:02	04:01	off therapy	719	≤50
EC6	01:01	24:02	38:01	57:01	06:02	12:03	ARV naive	1,219	≤50
EC7	01:01	34:02	08:01	57:01	06:02	07:01	off therapy	724	≤50
VC8	01:01	26:01	27:05	57:01	02:02	06:02	off therapy	357	105
EC9	03:01	26:01	15:01	27:05	02:02	03:03	ARV naive	871	≤50
EC10	02:01	03:01	15:01	27:05	01:02	04:01	ARV off	1,082	≤50

The analysis includes subject identifier (ID far left), detailed HLA subtypes, ARV therapy status, CD4 count (number/mm^3^), and viral load (copies/mL). CP, chronic progressor; EC, elite controller; VC, viremic controller; HLA, human leukocyte antigen; ARV, antiretroviral; VL, viral load.

As HIV particles are mainly composed of Gag and Pol proteins, we hypothesized that viral peptides presented through HLA-I shortly after HIV entry would preferentially target these proteins. We therefore tested whether the greater levels of recognition of non-productively infected cells in HIV controllers versus progressors (Figure 6A) were attributable to greater frequencies of HIV-Gag-specific CD8^+^ T cells. Most of the HIV progressor patients (9/10) showed CD8^+^ T cell responses to Gag peptide pool stimulation (final concentration of 1 μg/mL/peptide), as represented in Figure 6E (top), which was not significantly different from that of HIV controllers (Figure S5D). Following normalization of the IFN-γ response to non-productively infected cells to the total magnitude of Gag-specific CD8^+^ T cell response in the same subjects, we still observed proportionally greater responses in HIV controllers as compared to progressors (50% ±12% versus 18% ± 5.4%, p = 0.0334; Figure 6F). Of note, expression of programmed cell death protein 1 (PD-1), an exhaustion marker, was comparable in both groups (Figure S5C), and exhaustion blockade did not enhance cell killing (Figure S6). Thus, the increased responsiveness of CD8^+^ T cells from controllers to non-productively infected cells cannot be attributed to greater magnitude or differential exhaustion status of Gag-specific CD8^+^ T cell responses.

To further analyze the role of the CD8^+^ T cell response in HIV control, we evaluated whether there was a link between IFN-γ production by CD8^+^ T cells and the size of the reservoir in these patients, measured by digital droplet PCR *ex vivo,* as described by Henrich et al. (2012). Persons for whom HIV DNA could be measured were included (n = 6 for HIV progressors and n = 7 for HIV controllers). We observed a significant Spearman inverse correlation between the antiviral response and the size of the reservoir for all patients (Figure 6G, left), but also when only the HIV controllers were analyzed (Figure 6 G, right). Moreover, CD8^+^ T cells from HIV progressors did not respond to HIV-infected, non-activated CD4^+^ T cells, even with a blockade of exhaustion (Figure S6), which is consistent with a role for HIV-specific CD8^+^ T cells in containing the HIV reservoir in non-activated CD4^+^ T cells.

## Discussion

HIV reservoirs represent a major barrier to HIV eradication (Archin et al., 2014, Kimata et al., 2016), and substantial research efforts are being directed toward the goal of identifying and eliminating this obstacle to cure (Archin and Margolis, 2014, Pitman et al., 2018). Latently infected resting CD4^+^ T cells represent the majority of the HIV reservoir (Bruner et al., 2015, Siliciano and Siliciano, 2015), and it has been shown that HIV latency can be established in resting CD4^+^ T cells directly after infection, without intervening productive infection (Chavez et al., 2015). Therapeutic strategies aimed at eliminating HIV reservoirs have been dominated by the “kick and kill” paradigm, which involves reactivating latent CD4^+^ T cells to induce *de novo* production of viral proteins, and the subsequent elimination by the immune system, notably CD8^+^ T cells (Archin and Margolis, 2014, Pegu et al., 2015). Another strategy could be the elimination of infected cells before the establishment of the reservoir. Here, we show that *ex vivo* CD8^+^ T cells from HIV controllers (possessing at least one HLA-B^∗^27 or HLA-B^∗^57 allele), but not HIV progressors expressing the same alleles, are able to directly recognize and kill HIV-infected non-activated CD4^+^ T cells through the formation of immunologic synapses and class I restricted recognition of processed viral peptides. This occurs within a few hours following viral entry, allowing an antiviral response before HIV reverse transcription and thus before the eventual establishment of HIV latency. Moreover, this recognition precedes the production of the incomplete reverse transcripts that are needed to induce pyroptosis and the release of inflammatory molecules (Doitsh et al., 2010, Doitsh et al., 2014). These data indicate that HIV-specific CD8^+^ T cell responses have the potential to reduce the establishment of an HIV reservoir in non-activated CD4^+^ T cells and reduce HIV-induced inflammation, hence contributing to HIV control.

To further define the mechanism of recognition, we show that the recognition of non-productively infected resting CD4^+^ T cells by HIV-specific CD8^+^ T cells occurs through synapse formation and requires the presentation of viral peptides derived from incoming viral particles on HLA-I molecules. Moreover, we show that non-activated cells can be targeted either following exogenous infection or cell-to-cell spread, which is likely a major mode of ongoing viral replication *in vivo* (Chen et al., 2007, Dimitrov et al., 1993, Phillips, 1994). Our results indicate that incoming viral proteins are degraded in the cytosol of non-activated cells by the proteasome and the aminopeptidases to produce antigenic peptides for presentation by HLA class I, as blocking these enzymes or HLA class I reduced the CD8^+^ T cell responses.

It is important to note that these results were obtained using PBMCs from HIV controllers expressing HLA-B^∗^27 or HLA-B^∗^57 and with the laboratory strain of HIV NL4.3 possessing a wild-type sequence for most of the optimal epitopes. Further studies will be required to examine the recognition of non-activated infected cells in the context of other restricting HLA-I alleles. However, our results provide support for the hypothesis that proteins from incoming particles are degraded in the cytosol and presented directly at the surface of the target cell through HLA class I.

Previous studies have reported pre-integration presentation of the SIV-Gag peptides (Sacha et al., 2007) and the HIV-Gag KRWIILGLNK epitope on HLA-B^∗^27 in activated CD4^+^ T cells (Buseyne et al., 2001, Kløverpris et al., 2013). In addition, a previous study (Buckheit et al., 2013) performed with cells from HIV controllers possessing HLA-B^∗^57 reported the elimination of HIV^+^ resting CD4^+^ T cells by CD8^+^ T cells. The present study adds to these previous reports by using the Vpr-βlam assay and a GFP-expressing virus to precisely distinguish entry from protein production, by demonstrating that the observed effect is induced by the presentation of viral peptides on HLA-I molecules, by showing that functional immune synapses are required for recognition, and by demonstrating that more physiologic cell to cell transmission results in the sensitization of cells for CD8^+^ T cell recognition before the process of reverse transcription.

Antigen presentation of incoming viral particles has been well studied for diverse viruses in antigen-presenting cells (APCs), mostly in dendritic cells and macrophages in the context of the exogenous antigens’ pathway to prime the CD8^+^ T cells. However, CD4^+^ T cells are not known as efficient APCs, and thus the presentation of viral antigens from incoming particles through HLA-I that does not rely on *de novo* protein production is not completely expected and is not well characterized in CD4^+^ T cells. Furthermore, activated CD4^+^ T cells and resting CD4^+^ T cells possess different enzymatic activities regarding the antigen-processing pathway (J.B., unpublished data), and due to their activation status, resting CD4^+^ T cells are not expected to efficiently present antigen from incoming particles. Therefore, our study confirms that resting CD4^+^ T cells are able to present antigens from incoming particles through HLA-I and provides some mechanistic insight regarding this antigen-presentation pathway.

Additional studies will be required to understand why CD8^+^ T cells from HIV controllers are more efficient in recognizing non-activated infected cells and to determine factors that modulate this recognition. It will be important also to determine whether this is unique to the protective alleles HLA-B^∗^27 and B^∗^57 or can be seen in the context of other alleles. Nevertheless, these data indicate that non-productively infected cells can be targeted by CD8^+^ T cells, suggesting a path forward to reducing the viral reservoir. Furthermore, our study could have implications for T cell-based prophylactic vaccines as an adjunct to antibody-mediated vaccines, as the presence of these cells at the time of infection may limit the establishment of the reservoir.

## STAR★Methods

### Key Resources Table

**Table:** 

REAGENT or RESOURCE	SOURCE	IDENTIFIER
**Antibodies**		

AlexaFluor® 488 Phalloidin	Invitrogen	A12379; RRID:AB_2315147
AlexaFluor® 647 anti-CD107a clone H4A3	Biolegend	328612; RRID:AB_1227506
AlexaFluor® 647 anti-perforin clone dG9	Biolegend	308109; RRID:AB_493255
PE/CY7 anti-CD107a antibody clone H4A3	Biolegend	328617; RRID:AB_11147761
PE anti-CD3 clone HIT3a	Biolegend	300307; RRID:AB_314043
V500 anti-CD8 clone RPA-T8	BD Horizon	560775; RRID:AB_1937333
BV605 anti-CD4 clone OKT4	Biolegend	317437; RRID:AB_11204077
Pacific blue anti-Perforin clone B-D48	Biolegend	353305; RRID:AB_11124346
APC anti-IFN-γ clone B27	Biolegend	506510; RRID:AB_315443
FITC anti-TNF-α clone MAb11	BD PharMingen	554512; RRID:AB_395443
PerCP-Cy5.5 anti-MIP1-β clone D21-13551 RUO	BD PharMingen	560688; RRID:AB_1727567
AlexaFluor®700 anti-GranzymeB clone GB11	BD PharMingen	561016; RRID:AB_2033973
Purified anti-human CD11a clone 38	Genetex	GTX26132; RRID:AB_380799
Anti-human CD18 clone CBR LFA-1/2	Biolegend	366302; RRID:AB_2565276
Anti-human ICAM-2 clone CBRIC2/2	Genetex	GTX42521; RRID:AB_11169501
Anti-human LFA3 clone TS2/9	Biolegend	330912; RRID:AB_2075983
Ultra-LEAF™ purified anti- HLA-A, B, C	Biolegend	311428; RRID:AB_2561492

**Chemicals, Peptides, and Recombinant Proteins**		

Probenecid	Sigma-Aldrich	P8761
CellTracker™ Orange CMTMR dye	ThermoFisher	C2927
ProLong™ Gold Antifade mountant	ThermoFisher	P10144
GolgiStop™	BD Biosciences	554724
GolgiPlug™	BD Biosciences	555029
Fixable blue viability dye	Life Technologies	L-23105
BD Cytofix/Cytoperm™	BD Biosciences	554722
Efavirenz	Sigma	SML0536
Epoxomicin	Enzo Life Science	BML-PI127-0100
Bestatin	Sigma Aldrich	B8385
E64	Enzo Life Science	ALX-260-007
BsaJI	NEB	R0536L

**Critical Commercial Assays**		

CCF2-AM loading kit	Invitrogen	K1025
Dynabeads Human T-Activator CD3/CD28	ThermoFisher	111.31D
EasySep Human CD4+ T cell enrichment	Stemcell	19052
Alliance HIV-1 P24 ANTIGEN ELISA Kit	Perkin Elmer	NEK050001KT
Gentra Puregene cell kit	QIAGEN	158745

**Experimental Models: Cell Lines**		

HeLa cells	ATCC	CCL-2
HEK293T cells	ATCC	CRL-3216

**Recombinant DNA**		

Plasmid: NL4.3-GFP-X4	T.Murooka T. Mempel	N/A
Plasmid: NL4.3-GFP-R5	T.Murooka T. Mempel	N/A
Plasmid: pMM310	NIH	11444

**Software and Algorithms**		

IMARIS	Bitplane	http://www.bitplane.com/imaris
GraphPad Prism	GraphPad Software Inc.	https://www.graphpad.com/scientific-software/prism/
FlowJo	FlowJo LLC	https://www.flowjo.com/
Quantalife ddPCR software	QuantaLife, Inc.	http://www.bio-rad.com/

### Contact for Reagent and Resource Sharing

Further information and requests for reagents may be directed to and will be fulfilled by the Lead Contact, Bruce Walker (bwalker@mgh.harvard.edu).

### Experimental Model and Subject Details

#### Study subjects

PBMC from HIV-1-infected individuals were used for this study according to protocols approved by the Institutional Review Board of the Massachusetts General Hospital. Peripheral blood mononuclear cells (PBMC) were isolated using Ficoll-Hypaque density gradient centrifugation

HIV Controllers include Elite Controllers who were defined as having HIV-1 RNA below the level of detection for the respective available ultrasensitive assay (e.g., < 75 RNA copies/ml by cDNA or < 50 copies by ultrasensitive PCR) without antiretroviral therapy and Viremic Controllers as having HIV-1 RNA between 50 and 2000 RNA copies/mL. CD4+ T cell counts, viral loads and HLA types were determined as described (Pereyra et al., 2008). HIV Progressors were treated with Anti-retroviral (ARV) therapy for 1 to 4 years. Characteristics of the study subjects are shown in Table 1.

#### Cell lines

HeLa cells and HEK293T cells (both from ATCC) were cultured at 37°C in Dulbecco Modified Eagle Medium (DMEM) supplemented with 10% of Fetal Bovine Serum and 1% of penicillin/streptomycin.

### Method Details

#### Proviral DNA constructs

HIV proviral constructs coding for replicative NL4.3-GFP-X4 and NL4.3-GFP-R5 were previously described (Murooka et al., 2012) and were kindly provided by Thomas Murooka and Thorsten Mempel. Env-deleted (HIVΔEnv) and fusion-defective (HIV F522Y) HIV mutants were previously described (Bergeron et al., 1992, Clavel et al., 1989). F522Y carries a point mutation in Env, which abrogates fusion but retains CD4 binding ability.

#### Cells

Peripheral blood mononuclear cells (PBMC) were isolated using Ficoll-Hypaque density gradient centrifugation, rested a few hours at 37°C before being infected, checked for activity status or activated with anti-CD3/anti-CD28 beads. CD4+ T cells were isolated from PBMC by negative selection (StemCell Technologies, Vancouver, Canada). HeLa cells and HEK293T cells (both from ATCC) were cultured at 37°C in Dulbecco Modified Eagle Medium (DMEM) supplemented with 10% of Fetal Bovine Serum and 1% of penicillin/streptomycin.

#### HIV fusion assay (Vpr-βlam assay)

We relied on the previously described Vpr–β-lactamase (Vpr-βlam) assay (Cavrois et al., 2002, Tilton et al., 2014) to measure the efficiency of HIV entry into the cytosol of target cells, using cell-free virus preparations. Virus stocks were produced by co-transfecting 293T cells with HIV proviral clones and a plasmid encoding the *Vpr* gene fused to the *β-lactamase* gene (pMM310 NIH/AIDS Reagent program catalog # 11444). Virus preparations were concentrated by ultracentrifugation on 20% sucrose (1 hr, 22,000 rpm, 4°C). Primary non-activated PBMC or PBMC activated with anti-CD3/anti-CD28 immunomagnetic beads (Dynal, Invitrogen, ratio cell:beads 1:1) for 2 days, were then exposed to the virus preparation for 2 hours at 37°C. Cells were then washed and loaded with the CCF2-AM loading kit (Invitrogen) in the presence of 1.8 mM Probenecid (Sigma). Cells were incubated for 1 hr at room temperature and were then washed and fixed. Fluorescence following cleavage of CCF2-AM (excitation at 405 nm, emission at 450 nm) was measured by flow cytometry on a BD LSR-II system (Becton Dickinson) with FACSDiva8 software. These experimental conditions were then adapted to measure virus infection in the context of cell-cell contacts, as described (Monel et al., 2012). Briefly, HeLa cells were co-transfected with an HIV proviral clone and the Vpr–β-lactamase expressor plasmid. After 48 h, primary non-activated CD4+ T cells were added to the transfected and washed HeLa cells for 2 hours. The non-activated CD4+ T cells were then harvested, washed, and processed as described above for the cell-free virus assay. Target cells were gated based on their sizes to exclude doublets and syncytia.

#### Generation of HIV-specific CD8+ T cell lines

CTL lines specific were generated by stimulation with designated single peptides or a Gag peptide pool using cryopreserved PBMC from HLA-B^∗^27 or B^∗^57 HIV Controllers. PBMC were thawed and incubated with the designated peptide (final concentration 1ug/mL/peptide) for 10 days in R10 medium (RPMI supplemented with 10% fetal bovine serum plus HEPES buffer, penicillin, streptomycin, and L-glutamine). IL-2 (50 U/mL) was added on day 3. Specificity for cognate peptide was tested on day 10 by intracellular cytokine staining (ICS) for IFN-γ.

#### Confocal Microscopy and Analysis

Non-activated CD4+ T cells negatively isolated from PBMC were infected for 2 hours with HIV-X4 Vpr-βlam viruses. After infection, the cells were washed and incubated at 37°C. Two hours later the cells were loaded with the CCF2-AM loading kit for 1 hour at room temperature. During this time, autologous Gag pool-stimulated CD8+ T cell lines were incubated with 5M CellTracker™ Orange CMTMR dye according to the manufacturer’s instructions. CD4+ T cells and CD8+ T cells were then mixed together at a 1:1 ratio and were loaded onto polylysine-coated coverslips (1x10^6^ cells in 160uL) in 24 well plates. After 30 min at 37°C, cells were fixed with paraformaldehyde (PFA) 4% for 15min at room temperature, permeabilized with Triton 0.5% for 15min at room temperature, blocked with PBS containing 3% bovine serum albumin (BSA) for 1 hour at room temperature and stained with AlexaFluor® 488 Phalloidin (Molecular Probes) and AlexaFluor® 647 anti-CD107a (clone H4A3 Biolegend #328612) or AlexaFluor® 647 anti-perforin (clone dG9 Biolegend #308109) overnight at 4°C. The coverslips were then mounted on slides using ProLong Gold Antifade Reagent (Life Technology). Images were acquired with a Zeiss LSM 510 confocal microscope equipped with argon (488nm) and Diode (405nm, 561nm) lasers, 100X oil immersion objective and ZEN software. At least 10 contacts between CD4+ T cells and CD8+ T cells were analyzed per condition with Z stacks spaced by 0.33 μm. The 3D images were then reconstituted using the IMARIS software (Bitplane) and surfaces were created for each channel after background deduction.

##### Quantification of synaptic actin polarization

3D reconstituted images of synapses were analyzed with Imaris software. The fluorescent signal of the Phalloidin was quantified at the site of contact between the effector and the target cell and compared to the Phalloidin signal on the rest of the CD8+ T cell with Imaris software. Approximately 30 cells were analyzed per condition and the mean of the ratios (actin at point of contacts/actin in rest of CD8+ T cell) was calculated.

##### Actin ring quantification

3D reconstituted images of synapses between HIV+ non-activated CD4+ T cells and CD8+ T cells were analyzed with Imaris software. Surfaces were created for each channel after background deduction. En face views from each duplex were taken and the blue pixels (target cells) were removed to allow an unobstructed view of the actin stain at the interface. The percentage of cells showing a surface corresponding to actin in a ring-shape was blindly determined for each experiment.

##### Perforin and CD107a quantification at the site of contact

3D reconstituted images of synapses were analyzed with Imaris software. A 3D rectangle was drawn to delimit the interface between the two cells. Spots objects identifying point-like objects were automatically created using the “Spots” tool of the Imaris software for the channel corresponding to CD107a or perforin staining after background deduction. The percentage of interfaces presenting perforin or CD107a spots was determined for each experiment.

#### Flow cytometry analysis

Non-activated PBMC were infected or not with the indicated HIV strains for 2 hours at 37°C. Free virions were then washed with PBS. The cells were resuspended in fresh media and incubated 5 hours at 37°C in the presence of PE/CY7 anti-CD107a antibody (clone H4A3 Biolegend #328617) and in the presence of GolgiStop™ (BD Biosciences #554724) and GolgiPlug™ (BD Biosciences #555029) for intracellular cytokine staining. The cells were then harvested and stained for viability (Fixable blue viability dye, Life Technologies #L-23105) for 30 min, then stained with PE-CD3 (clone HIT3a Biolegend #300307), V500-CD8 (clone RPA-T8 BD horizon #560775) and BV605-CD4 (clone OKT4 Biolegend #317437). After fixation and permeabilization (BD Cytofix/Cytoperm™ #554722) the cells were stained for Pacific blue-Perforin (clone B-D48 Biolegend #353305), APC-IFN-γ (clone B27 Biolegend #506510), FITC-TNF-α (clone MAb11 BD PharMingen #554512), PerCP-Cy5.5-MIP1-β (clone D21-13551 RUO BD PharMingen #560688) and AlexaFluor^®^ 700-GranzymeB (clone GB11 BD PharMingen #561016). Fluorescence signals were then quantified using a BD LSR-II flow cytometry system (Becton Dickinson) with FACSDiva8 software and results were analyzed with FlowJo software.

#### Blocking antibodies and drugs

For blockade of adhesion molecules, purified anti-human CD11a antibody (Genetex^®^ cat: GTX26132 clone: 38), anti-human CD18 antibody (Biolegend^®^ cat: 366302 clone CBR LFA-1/2), anti-human ICAM-2 antibody (Genetex^®^ cat: GTX42521 clone: CBRIC2/2) and anti-human LFA3 antibody (Biolegend^®^ cat: 330912 clone: TS2/9) were used.

For blockade of the HLA class I molecule, Ultra-LEAF™ purified anti-human HLA-A, B, C antibody (BioLegend^®^ clone W6/32) was used. In order to block HIV reverse transcription, the retroviral drug Efavirenz (Sigma; SML0536) was used at a final concentration of 100nM. For the study of antigen processing pathways, the proteasome hydrolytic activities were blocked with epoxomicin (Enzo Life sciences-BML-PI127-0100), the aminopeptidase activities with Bestatin (Sigma-Aldrich B8385) and the cysteine proteases (cathepsins B, H and L) with E64 (Enzo Life Sciences ALX-260-007) at different final concentrations as indicated on the figures.

#### HIV cell-to-cell transmission

HIV cell-to-cell transmission was performed as previously described (Monel et al., 2012). Donor HeLa cells were transfected with different proviral constructs (HIV X4, HIV R5, HIV F522Y) and plated in 24-well plates (10^5^ cells per well). Cells were then washed to eliminate cell-free virions 48 h post-transfection, and 5x10^5^ target cells (non-activated CD4+ T cells) were added in a final volume of 500 μl. Target cells were collected 2 hours later; an aliquot was used for a Vpr-βlam assay to check HIV entry while remaining cells were co-cultured (ratio 1:1) with autologous CD8+ T cells in 24-well plates for 5 hours in the presence of anti-CD107a antibody. Live CD8+ T cells were then analyzed by flow cytometry for degranulation.

#### Chromium release assay

Primary non-activated CD4+ T cells were isolated from PBMC by negative selection (EasySep™ Human CD4+ T cell enrichment kit from StemCell) and infected with 500ng of p24 (determined by ELISA) of NL4.3 containing the fusion protein Vpr-βlam for 2 hours at 37°C. After infection, the cells were washed, and an aliquot was removed to perform an HIV fusion assay as described ahead. The remaining cells were labeled with chromium (around 50 μCi) for 1 hr at 37°C. HIV Gag pool- or Gag-KK10-stimulated CD8+ T cell lines were then added at the indicated effector-target ratios, and a standard 6 hour chromium release assay was performed as previously described (Yang et al., 2003). The plates were spin down to pellet the cells and the supernatants were transferred from each well to the well of an absorbent plate. The plates were dried overnight and put in a plate reader to measure the ^51^Cr release. Each sample were performed at least in duplicate. Percent specific lysis was calculated as [(mean experimental cpm – mean spontaneous cpm)/(mean maximum cpm – mean spontaneous cpm)] × 100. Spontaneous and maximum releases were determined by incubating the labeled target cells with medium alone or 5% Triton X-100, respectively.

#### Quantification of HIV DNA reservoir by digital droplet PCR

Digital droplet PCR was performed as previously described (Henrich et al., 2012). Genomic DNA was extracted from primary non-activated CD4+ T cells using the Gentra Puregene kit (Gentra) following the manufacturer’s instructions. For each PCR reaction, 5 units of restriction enzyme BsaJI (NEB) was directly mixed with 300ng of DNA, ddPCR Supermix for Probes (Bio-Rad), and final concentrations of 900nM primers and 250nM probe. Primers/Probes were: RPP30 – forward primer GATTTGGACCTGCGAGCG, reverse primer GCGGCTGTCTCCACAAGT, probe VIC-CTGAACTGAAGGCTCT-MGBNFQ; HIV-gag – forward primer TACTGACGCTCTCGCACC, reverse primer TCTCGACGCAGGACTCG, probe FAM-CTCTCTCCTTCTAGCCTC-MGBNFQ. Droplets were prepared using the QX100 Droplet Generator (Bio-Rad) following the manufacturer’s instructions. Sealed plates were cycled using the following program: 95°C for 10 min; 40 cycles of 94°C for 30 s, 60°C for 1 min; and 98°C for 10 min, with 2°C/sec ramping speed to ensure even droplet heating. Reactions were analyzed using the QX100 Droplet Reader and number of template molecule per μl of starting material was estimated using the Quantalife ddPCR software. We aimed to run 8 replicates of ddPCR per sample – depending on the amount of DNA availability. We consistently applied a pre-determined exclusion criterion to outliers that deviated from mean values by > 2x the standard deviation.

### Quantification And Statistical Analysis

Most of the data are represented as Mean ± standard deviation and statistical analyses were performed using GraphPad PRISM software. t tests were used to compare two groups of samples, one-way ANOVA multiple comparison tests were used to compare each column to a control column or to every other columns and two-way ANOVA multiple comparison tests were used when grouped data were analyzed and compare to a control column (Figures 5B and 5C). Regarding Figure 6G we used a Spearman correlation to assess monotonic relationships between the Log_10_ of Gag copies/10ˆ6 non-activated CD4+ T cells and the CD8+ T cell IFN-γ response. The Spearman’s rank correlation coefficient (r_s_) is indicated in the Figure. The corresponding tests are indicated for each figure in the legend. In Figure 6, the n indicated in the legend represents the number of patients studied for each group (n = 10 Controllers and n = 10 Progressors for Figures 6A–D and 6F; n = 12 for Figure 6 left panel; n = 6 Controllers and n = 6 Progressors for Figure 6G right panel. No methods were used to determine whether the data met assumptions of the statistical approach.

## Supplemental Information

Document S1. Figures S1–S6

Document S2. Article plus Supplemental Information
