# Supplementary material for: HIV Controllers Exhibit Effective CD8+ T Cell Recognition of HIV-1-Infected Non-activated CD4+ T Cells
Source: Cell Rep. 2019 Apr 2;27(1):142–153.e4. doi: 10.1016/j.celrep.2019.03.016 (PMC6449512; doi:10.1016/j.celrep.2019.03.016)
Supplement: Document S1. Figures S1–S6 [file mmc1.pdf]

Cell Reports, Volume *27*

Supplemental Information

HIV Controllers Exhibit Effective CD8$^+$ T Cell Recognition of HIV-1-Infected Non-activated CD4$^+$ T Cells

Blandine Monel, Annmarie McKeon, Pedro Lamothe-Molina, Priya Jani, Julie Boucau, Yovana Pacheco, R. Brad Jones, Sylvie Le Gall, and Bruce D. Walker

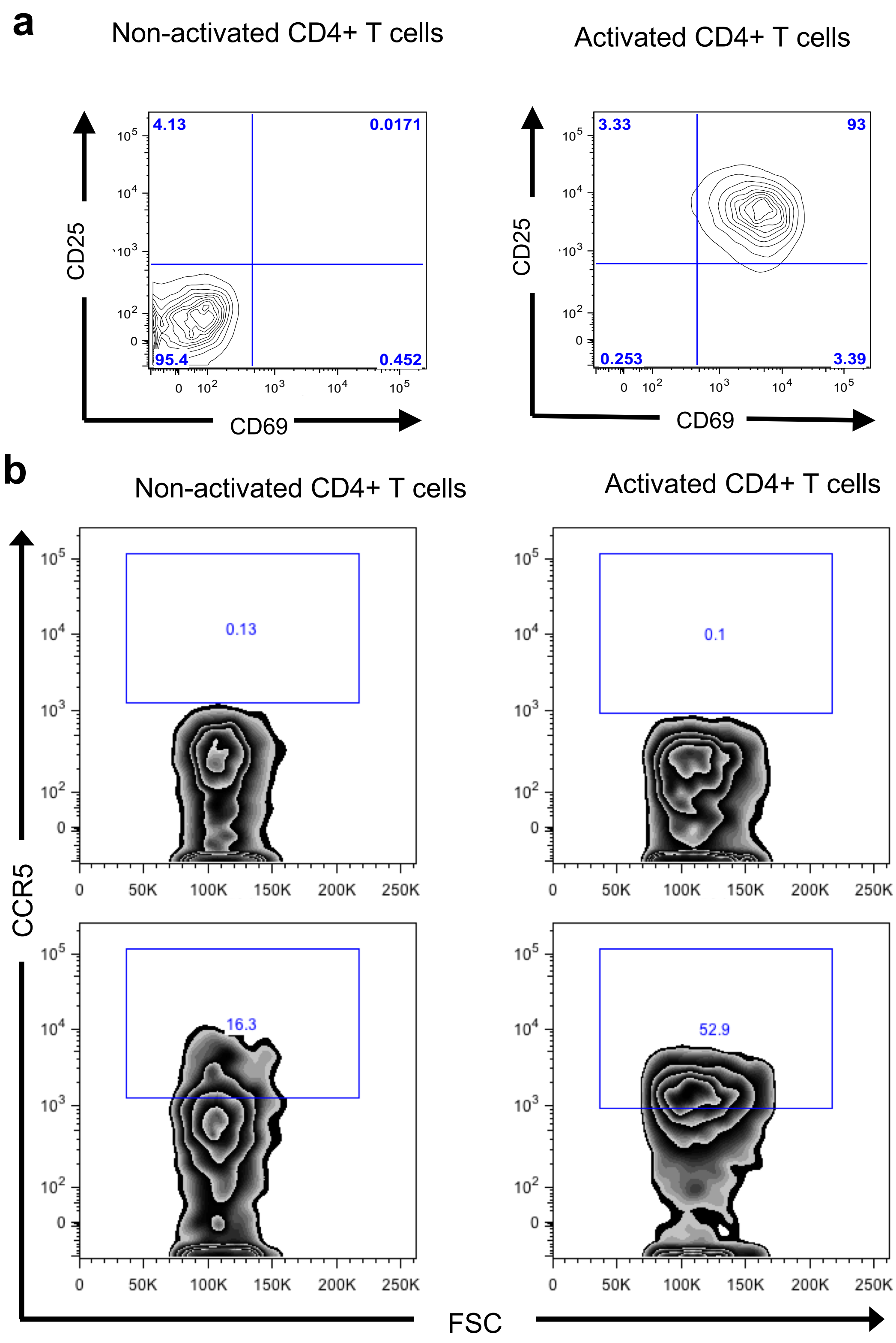

Figure S1. Characterization of primary non-activated and activated CD4+ T cells, related to Figure 1. (**a**) Primary non-activated CD4+ T cells were identified by the absence of CD25+/CD69+ expression on CD4+ T cells, whereas in vitro stimulation with anti-CD3 and anti-CD28 lead to expression of both markers. **(b)** The frequency of primary CCR5+ CD4+ T cells was measured by flow cytometry before (left panel) or after 2 days of activation with CD3/CD28 beads (right panel) and compared to isotype controls (upper panel)**.**

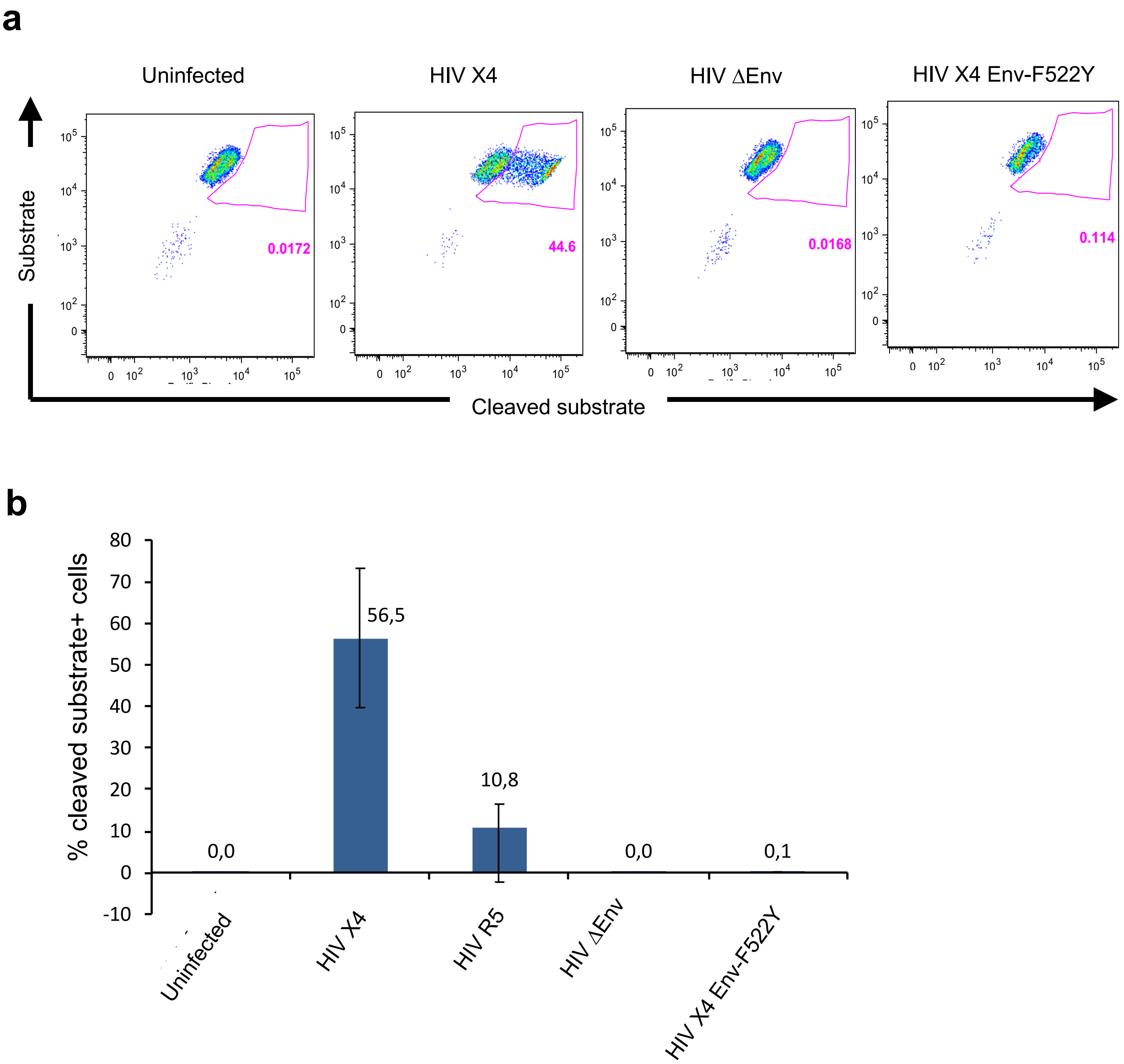

Figure S2. HIV infection in primary non-activated CD4+ T cells, related to Figure 1. Non-activated CD4+ T cells were infected for 2 hours with NL4.3 X4 (HIV X4), a virus missing the envelope (HIV ΔEnv) or a fusion defective virus (HIV X4 Env-F522Y) all carrying the fusion protein Vpr-β lactamase and an IRES-GFP cassette. HIV entry was determined 2 hours later by incubation with a FRET β-lactamase substrate, where cleaved substrate+ cells represent HIV+ cells. A representative experiment is shown **(a)** and the results from three independent experiments with cells coming from three different HIV Controllers patients are shown as mean ± standard deviation (**b**).

a

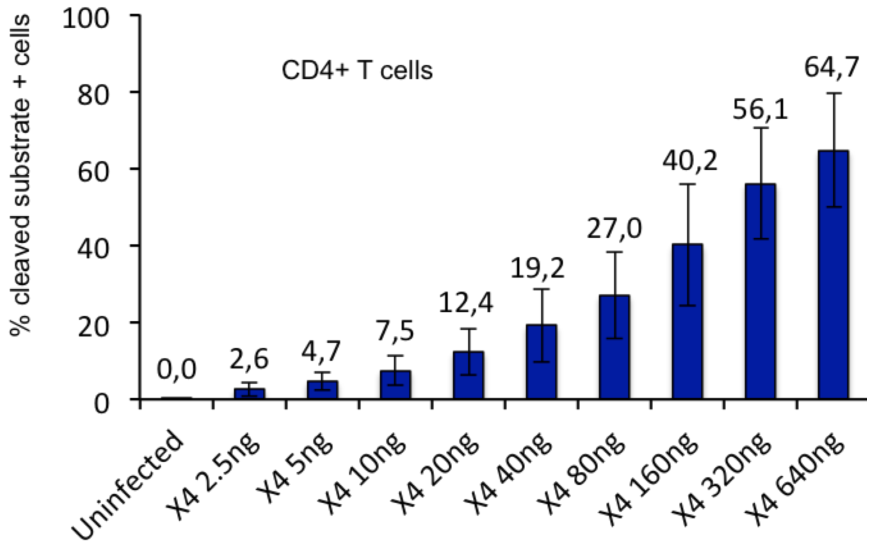

b

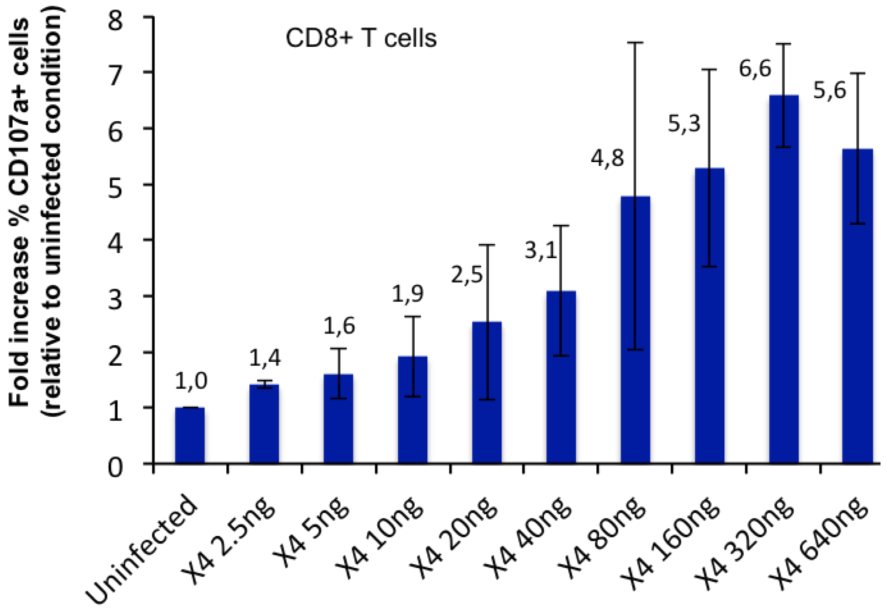

Figure S3. Viral doses experiment, related to Figure 2. Non-activated CD4+ T cells were infected for 2 hours with different amount of HIV X4 (as quantified by p24 ELISA) and a Vpr-βlam assay was performed **(a).** The CD8+ T cell response was then quantified 5 hours later by measuring CD107a expression **(b).** The results are shown as means ± standard deviations for three independent experiments from three different individuals.

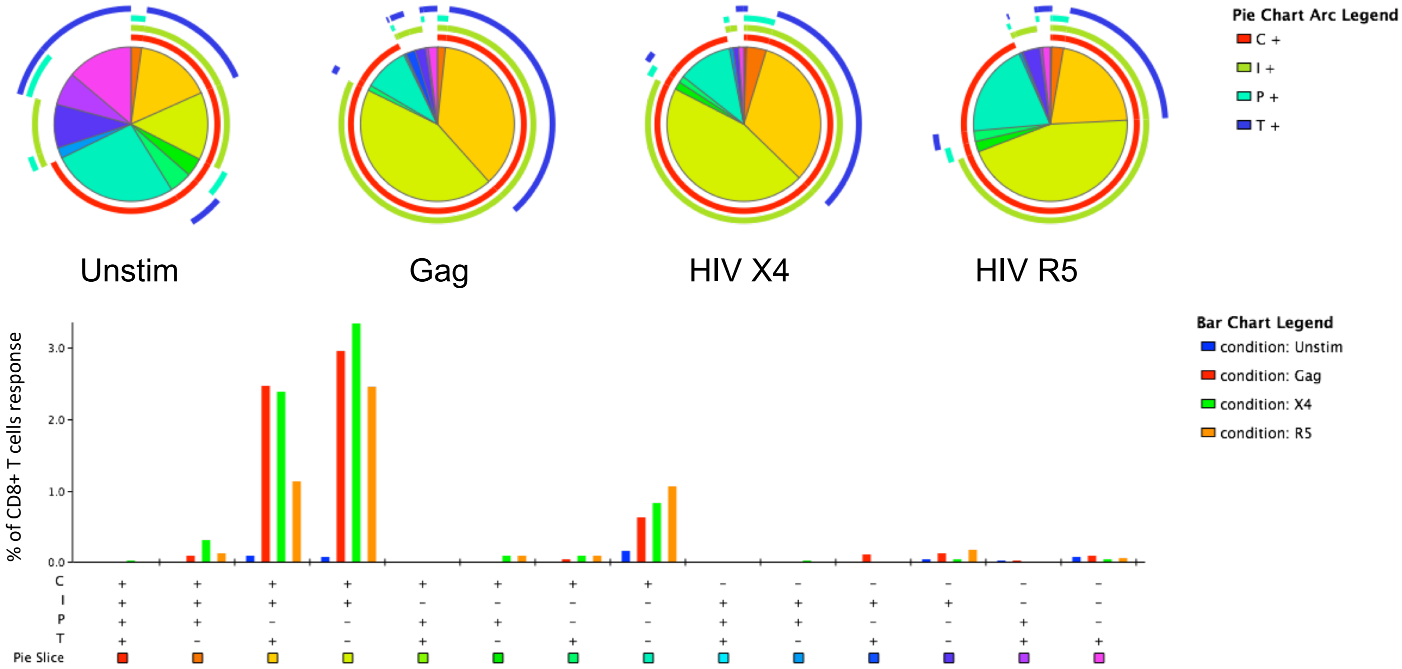

Figure S4. Polyfunctionality profile of the CD8+ T cells response to HIV+ autologous non-activated CD4+ T cells versus Gag peptides loaded CD4+ T cells targets, related to Figure 2. Activation of CD8+ T cells was measured by intracellular cytokine staining (IFN-g, Perforin, TNF-a) in addition to CD107a staining and by flow cytometry. The polyfunctionality profile was created with Spice software.

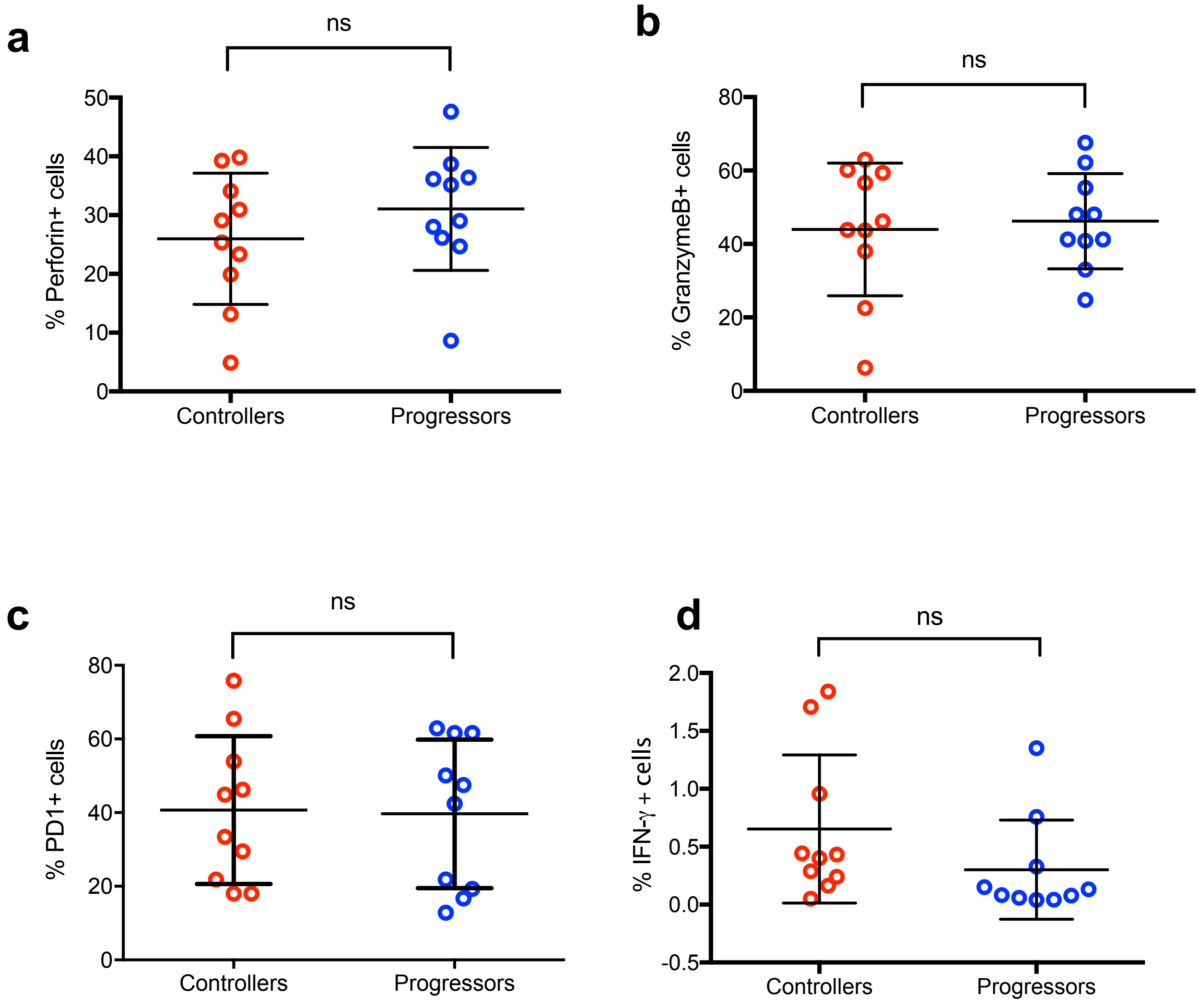

Figure S5. Characterization of CD8+ T cells from HIV Controllers versus Progressors, related to Figure 6. **(a)** Perforin expression**.** **(b)** Granzyme B expression**.** **(c)** PD1 expression**.** **(d)** CD8+ T cell response to Gag pool peptides stimulation.

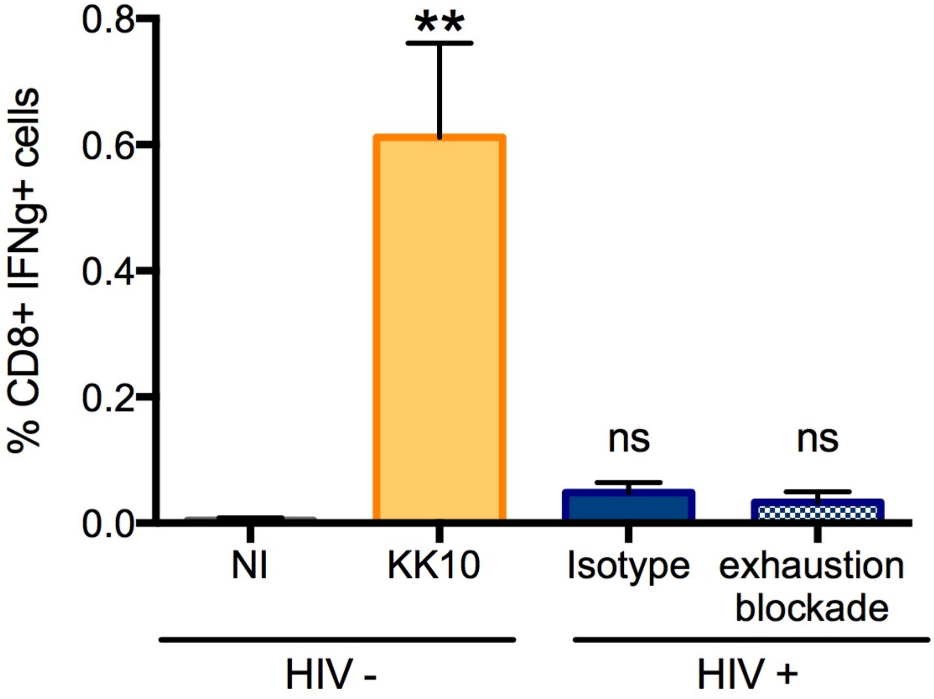

Figure S6. Characterization of CD8+ T cells response from HIV Progressors with an exhaustion marker blockade, related to Figure 6. CD8+ T cell response in 3 HIV Progressors was analyzed by intracellular staining of IFN-γ and flow cytometry after stimulation with KK10 peptide or HIV infection in presence or not of blocking peptides to exhaustion markers (PDL-1, TIM3 and 2B4)**.** Means ± standard deviations and statistics were calculated with ANOVA multiple comparison test relative to Non-infected condition (NI) condition ** $p<0.01$.
